# Prevalence and Risk Factors of Gastrointestinal Parasitic Infections in Buffaloes Across Commercial and Subsistence Farming Systems in Butwal, Nepal

**DOI:** 10.1155/vmi/4985465

**Published:** 2026-06-02

**Authors:** Arti Neupane, Shristi Bhandari, Tikaram Khanal, Kishor Pandey, Rajendra Prasad Parajuli

**Affiliations:** ^1^ Central Department of Zoology, Tribhuvan University, Kritipur, Kathmandu, Bagmati, Nepal, tribhuvan-university.edu.np; ^2^ Department of Livestock Services, Central Referral Veterinary Hospital, Ministry of Agriculture and Livestock Development, Government of Nepal, Tripureshwor, Kathmandu, Bagmati, Nepal; ^3^ Herbert Wertheim School of Public Health and Human Longevity Science, University of California San Diego (UCSD), San Diego, California, USA, ucsd.edu

**Keywords:** buffaloes, gastrointestinal diseases, livestock, Nepal, parasitic diseases, risk factors

## Abstract

**Background:**

Gastrointestinal parasites (GIPs) impair livestock productivity and impose economic burdens on farmers. Buffaloes are central to Nepal’s dairy sector, yet comparative data on GIP burden across farming systems remain limited.

**Objective:**

This study investigates GIP prevalence and associated risk factors in buffaloes under commercial and subsistence farming systems in Butwal submetropolitan city, Nepal, an area of rapidly expanding dairy production.

**Methods:**

From July to September 2023, 224 fecal samples (112 per farming system) were collected from female buffaloes aged ≥ 3 years. Samples were examined microscopically using flotation and sedimentation techniques, and management and environmental factors were captured through structured questionnaires and field observations. Logistic regression identified predictors of infection.

**Results:**

Overall, 58.9% of samples contained oocysts or eggs of one or more GIP. The identified parasites included three protozoa (*Entamoeba* spp., *Balantidium* spp., and coccidian), three nematodes (*Strongyloides* spp., *Toxocara* spp., and *Haemonchus* spp.), and three trematodes (*Fasciola* spp., *Paramphistomum* spp., and *Schistosoma* spp.). *Fasciola* spp. (30.8%) and *Entamoeba* spp. (26.3%) were most prevalent. Commercial farms had significantly higher nematode infections (10.7% vs. 2.7%, *p* = 0.029), while subsistence farms showed slightly higher protozoan infections (33.9% vs. 25.9%). Free‐ranging rearing (aOR = 3.11, 95% CI: 1.15–8.43) and irregular health checkups (aOR = 2.92, 95% CI: 1.05–8.12) were strongly associated with infections. The presence of other free‐range animals and wallowing also influenced infection patterns.

**Conclusion:**

The substantial GIP burden and system‐specific risk factors highlight the need for improved parasite management. Enhanced deworming, better grazing practices, and farmer education may help reduce infections and improve buffalo productivity.

**Highlights**
• The GIP prevalence in buffaloes was 58.9% across farming systems in Butwal, Nepal.
• *Fasciola* spp. (30.8%) and *Entamoeba* spp. (26.3%) were the most common parasites.
• Commercial farms had more nematodes, and subsistence farms had more protozoan infections.
• Free‐ranging rearing increased the GIP infection risk (aOR 3.11; 95% CI: 1.15–8.43).
• Irregular health checks strongly predicted GIP infections (aOR 2.92; 95% CI: 1.05–8.12).
 Prevalence and risk factors of gastrointestinal parasitic infections in Buffaloes across commercial and subsistence farming systems in Butwal, Nepal

## 1. Introduction

Buffaloes have long been domesticated in Nepal and India for milk and meat production, and buffalo farming has now expanded globally. In Nepal, the growing demand for buffalo dairy and meat products has led to the rapid expansion of commercial buffalo farming, attracting increasing numbers of entrepreneurs to the dairy sector. Nepal hosts an estimated 5.16 million buffaloes, with Lumbini Province alone accounting for 23.95%, the highest concentration nationwide [1]. Buffaloes contribute substantially to the national economy by supplying milk, meat, and compost manure [2]. Agriculture represents 24.12% of Nepal’s agricultural gross domestic product (AGDP), and the livestock sector contributes approximately 24.01% of the AGDP. Specifically, buffalo milk accounts for 7.28% of AGDP, whereas buffalo meat contributes an additional 4.23% [1]. With this rising demand for dairy and meat, many farmers are increasingly encouraged to adopt livestock farming, ranging from small‐scale subsistence systems to larger commercial operations. Smallholder farmers in rural areas typically rear one to three buffaloes, relying on locally available feed resources such as grasses and crop residues and allowing animals to graze freely in open fields, forests, and riverbanks. This traditional system supports household subsistence with minimal external inputs. In contrast, some smallholder and commercial farmers raise buffaloes in confined or semiconfined systems, supplying all feed and fodder and restricting free grazing—a less common but increasingly adopted approach among commercial producers seeking higher efficiency and productivity.

Gastrointestinal parasites (GIPs) in buffaloes cause inflammation of the intestinal mucosa, leading to indigestion, malabsorption, diarrhea, and competition for nutrients [2]. These effects contribute to reduced growth, lower milk productivity [3], and ultimately economic losses for farming communities [4]. Globally, buffaloes are affected by a diverse range of GIP, including trematodes (*Fasciola* spp., *Paramphistomum* spp., *Schistosoma* spp.); nematodes (*Dictyocaulus viviparus*, Strongylidae, *Haemonchus* spp., *Cooperia* spp., *Ostertagia* spp., *Trichostrongylus* spp., *Oesophagostomum* spp.); and protozoa (*Eimeria* spp., *Buxtonella sulcata*, *Entamoeba* spp., *Balantidium* spp., *Cryptosporidium* spp.) [2]. Prevalence estimates vary widely across regions, from 35% [5] to 100% [6, 7], including reports from countries such as Malaysia [3] and China [8].

The factors associated with higher GIP burden include frequent free‐grazing practices [9], inconsistent administration of anthelmintics [10], and rearing styles [11, 12]. Additionally, failure to conduct routine health checkups increases susceptibility to gastrointestinal infections [11]. The prevalence of GIP is strongly influenced by ecological conditions, including proximity to rivers [13], temperature [14], humidity, precipitation [3], altitude, and the existence of open sewage near pasturelands [15]. In addition to these broader environmental factors, the farm‐level microenvironment, such as the source and quality of grass, water availability, and sanitation of animal sheds, has also been shown to contribute to variations in GIP prevalence [16].

The tropical climate of the study area, which has high temperatures, high humidity, and generally poor sanitation, creates favorable conditions for parasite proliferation. In this region, farmers increasingly prefer buffaloes over cows because of several economic and cultural advantages. Buffalo milk commands a higher market price than cow milk does [1], and once buffaloes become old, nonlactating, infertile, or difficult to breed, they can still be sold at high value for meat—an option not available for cows in many Hindu communities in Nepal. Buffaloes are also considered easier to rear and less prone to frequent illness, and both male and female offspring have substantial economic value, particularly male buffaloes for meat. Consequently, the number of buffalo farms in both commercial and subsistence systems has risen steadily. In addition, compared with cows, buffaloes tend to wallow in muddy water, rest in wet or contaminated areas, and often use common water sources, which may increase their exposure to environmental parasites.

Despite this growing trend, no studies have evaluated the prevalence of GIPs in buffaloes raised under commercial versus subsistence farming systems in the Rupandehi district. Unlike prior Nepalese studies that were primarily descriptive, our study is the first to quantify the adjusted effects of farm‐ and host‐level factors on buffalo GIP using multivariable regression, thereby addressing confounding factors and establishing independent associations beyond simple prevalence comparisons (including across farming systems). With the rising demand for buffalo milk and the dual economic benefit of dairy production followed by meat sales, the submetropolitan city has experienced rapid expansion of buffalo farming. Given these developments, assessing the burdens of GIP and identifying associated risk factors across different farming systems are essential. Therefore, this study was carried out to determine the prevalence of GIP in buffaloes and examine how commercial and subsistence farming practices influence infection patterns in Butwal submetropolitan city, Nepal.

## 2. Materials and Methods

### 2.1. Study Area

**FIGURE 1:**
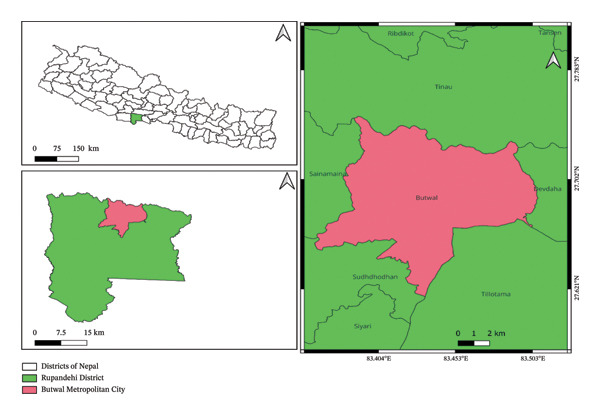
Map of the study area.

The study was conducted in Butwal submetropolitan city, which is located in the Rupandehi District of Lumbini Province in the midwestern region of Nepal (Figure 1). Butwal covers an area of approximately 101.61 km^2^ and is administratively divided into 19 wards, with a total population of 195,054 (Central Bureau of Statistics) [17]. The city lies along the Tinau River at the northern edge of the Terai plains, just below the Siwalik Hills, and is characterized by flat terrain with an elevation ranging from 130 to 150 m above sea level (coordinates: 27.70°N, 83.46°E). Although Butwal is primarily a commercial and trading hub, agriculture and livestock rearing remain important livelihoods for many residents. Buffaloes and cows are raised both on commercial farms within the city and on smaller subsistence farms located in surrounding villages. These mixed production systems provide a suitable setting for examining GIP infections under differing management and environmental conditions. The study was conducted during July–September, the postmonsoon period in a humid subtropical climate characterized by high temperature, high humidity, and extensive open grazing areas that influence parasite survival and transmission.

### 2.2. Study Design and Sample Collection

A purposive sampling approach was applied. Farms with ≥ 10 buffaloes, formally registered with the municipality and engaged in commercial trading, were classified as commercial farms. Farms with < 10 buffaloes, not registered, and maintained primarily for household subsistence were classified as subsistence farms. Male buffaloes were excluded because very few were kept in the study area, and sampling only females ensured uniformity in age, management, and production characteristics across farms. Only female buffaloes aged ≥ 3 years and not treated with anthelmintics within the preceding 3 months were eligible. For commercial farms, only those maintaining updated health records were included. Buffaloes < 3 years old, males, recently dewormed animals (< 3 months), and commercial farms lacking health records were excluded. Commercial farms were purposively identified using local veterinary and livestock extension records, based on predefined eligibility criteria such as herd size, active production status, and willingness of farm owners to participate. A formal sample size or power calculation was not performed. The study sample was determined pragmatically based on logistical feasibility, farm availability, and time constraints inherent to the student‐led nature of the study. This study therefore represents a selected population of adult female buffaloes meeting predefined inclusion criteria, and findings should be interpreted within this context.

Data collection was conducted between July and September 2023. Written informed consent was obtained after the farm owners were briefed about the study objectives and procedures. A total of 224 fecal samples were collected from nine wards (Wards 11–19) of Butwal submetropolitan city—112 from commercial farms and 112 from subsistence farms. For commercial farms, 16 farms were selected: two farms from each of seven wards and one farm each from Wards 11 and 17 due to limited availability. From each commercial farm, seven freshly voided dung samples were collected, resulting in 112 samples. For subsistence farms, seven households located near each of the 16 commercial farms were approached, and one freshly voided sample was collected per household to ensure an equal number of samples for comparative analysis. Subsistence farms were selected near commercial farms to ensure comparable microecological conditions and minimize environmental confounding between production systems.

Approximately 10–15 g of fresh fecal material was collected immediately after defecation, taking care to avoid contamination with soil, urine, or bedding by using gloves and a sterile spatula. The samples were then transferred into screw‐capped vials and preserved in 2.5% potassium dichromate until laboratory examination. The authorization to collect fecal samples was granted by the Nepal Veterinary Council Ethics Committee (Approval No: 82/2080/81). Informed consent was obtained from all farm owners before sample collection, with assurances of confidentiality, anonymity, and the right to withdraw at any time without consequences. Prior to sampling, the buffalo owners were verbally informed about the study’s purpose and reassured that the procedures would not harm their animals. Fecal samples were collected under strict hygienic conditions, and all specimen handling and collection procedures followed relevant institutional and national guidelines. No animals were harmed during the sampling process.

### 2.3. Questionnaire Survey

During the field visit and sample collection, personal interviews were conducted with farm owners or caretakers from both commercial farms and subsistence farms. A structured questionnaire was used to gather sociodemographic information on farm management and health management practices (Table 1) to identify and evaluate potential risk factors associated with GIP infections. Buffalo breed classification followed farm‐owner and veterinary records, and self‐raised animals could fall into either Murrah or cross‐bred categories. Bedding type was recorded as reported by farmers, where “edible bedding” referred to materials buffaloes commonly consume (e.g., straw, hay), and “non‐edible bedding” included materials they do not eat (e.g., sand, sawdust). Roof type was included as a proxy indicator of overall shed quality and farm financial capacity, which are indirectly linked to hygiene, drainage, and parasite exposure risk. Other water sources referred to wells, ponds, rivers, and stream water used for livestock drinking and cleaning.

**TABLE 1:** Characteristic and farm management features of female buffalo at commercial and subsistence farms (*n* = 224).

Characteristics	Commercial (*n* = 112)	Subsistence (*n* = 112)	*p* value	Total
Demographic characteristics	Mean (SD)/*n* (%)	Mean (SD)/*n* (%)		Mean (SD)/*n* (%)
Age (in years)	7.38 (1.82)	6.48 (2.01)	< 0.001^$^	6.93 (1.97)

*Other characteristics*				
*Breed of buffalo*				
Murrah	16 (14.3)	0 (0)	< 0.001^#^	16 (7.1)
Crossbred	96 (85.7)	112 (100)		208 (92.9)

*Source of livestock*				
Self‐raised	10 (8.9)	72 (64.3)	< 0.001^∗^	81 (36.3)
Bought from traders	102 (91.1)	40 (35.7)		142 (63.4)

*Type of shed*				
Stall barn	105 (93.8)	112 (100)	< 0.05^#^	217 (96.9)
Loose housing	7 (6.3)	0 (0)		7 (3.1)

*Flooring and roofing of shed*				
Concrete floor and tin roof	105 (100)	105 (93.8)	< 0.05^#^	210 (96.8)
Earth floor and tin roof	0 (0)	7 (6.3)		7 (3.2)

*Use of bedding*				
Nonedible bedding	112 (100)	35 (31.3)	< 0.001^#^	147 (65.6)
Edible bedding	0 (0)	77 (68.8)		77 (34.4)

*Present condition of buffalo*				
Pregnant	63 (56.3)	57 (50.9)	> 0.05^∗^	120 (53.6)
Milking	49 (43.8)	55 (49.1)		104 (46.4)

*Free range*				
No	84 (75)	70 (62.5)	> 0.05^∗^	154 (68.8)
Yes	28 (25)	42 (37.5)		70 (31.3)
Mode of feeding grass				
Cut and carry	112 (100)	112 (100)	> 0.05^∗^	224 (100)
Grazing	0 (0)	0 (0)		0 (0)

*Wallowing*				
No	91 (81.3)	81 (72.3)	> 0.05^∗^	172 (76.8)
Yes	21 (18.8)	31 (27.7)		52 (23.2)

*Source of water*				
Tap water	105 (93.8)	112 (100)	< 0.05^#^	217 (96.9)
Other sources	7 (6.3)	0 (0)		7 (3.1)

^$^Independent *t* test.

^∗^Chi‐square test.

^#^Fisher’s exact test when any cell has a count less than 5.

### 2.4. Examination of Fecal Samples and Identification of GIP

The samples were examined via direct wet‐mount preparation, followed by flotation and sedimentation techniques. Parasite ova, cysts, oocysts, and larvae were identified on the basis of their characteristic morphology, including shape, size, and color, via iodine wet mounts and concentrated preparations from flotation and sedimentation methods, following standard diagnostic criteria [18]. Parasite stages were measured using a compound microscope equipped with a calibrated eyepiece micrometer.

#### 2.4.1. Direct Wet Mount

Approximately two spatulas of well‐mixed fecal material were used to prepare direct smears. A drop of the sample with and without Lugol’s iodine was placed on separate glass slides, covered with cover slips, and examined microscopically under 10× and 40× magnifications [18].

#### 2.4.2. Concentration Technique

Because parasite stages such as eggs, cysts, and trophozoites are often present in very small quantities in the fecal material, they are not reliably visible through direct smear examination. To enhance detection, various concentration procedures were employed, specifically the saturated salt flotation method and the formalin–ether (FE) sedimentation technique [18]. Flotation was used to isolate the lighter nematode and cestode eggs, whereas sedimentation was applied to recover the denser trematode eggs. The concentrated preparations were then inspected under a microscope at 10× and 40× magnifications, with identification on the basis of characteristic morphological features [18].

#### 2.4.3. Saturated Salt Flotation

The flotation technique relies on the principle that small and lightweight nematodes and cestode eggs rise to the surface in a high‐density solution. For this procedure, approximately two spatulas of fecal material were stained and transferred into a 15‐mL centrifuge tube and then thoroughly mixed with 12 mL of 0.9% w/v sodium chloride (NaCl). The mixture was subsequently centrifuged at 1200 rpm for 5 min, after which the supernatant was discarded. The pellet was subsequently resuspended in 45% w/v NaCl and centrifuged again at the same speed for the same duration. After centrifugation, the tube was topped with saturated NaCl solution and allowed to stand undisturbed for 15 min, with a coverslip placed gently over the rim to collect floating eggs. The coverslip was then carefully lifted, mounted onto a glass slide, and examined microscopically [19].

#### 2.4.4. FE Sedimentation

Compared with other helminth eggs, trematode eggs are denser and therefore do not rise in flotation solutions, making sedimentation the preferred method for their detection. In this procedure, approximately two spatulas of fecal material were mixed thoroughly with 12 mL of 0.9% w/v sodium chloride (NaCl) in a 15‐mL centrifuge tube and centrifuged at 1200 rpm for 5 min. The supernatant was discarded, after which 10 mL of 10% formalin and 3 mL of ether were added. The tube was subsequently centrifuged again under the same conditions. Following removal of the upper layers, a small portion of the remaining sediment was transferred onto a clean glass slide using a pipette and examined microscopically [20].

#### 2.4.5. Identification

After examination of both the stained and unstained fecal preparations at 10 × and 40×  magnifications, egg and cyst dimensions were measured using an Olympus C × 40 trinocular microscope [21]. Parasite eggs, oocysts, and larvae were identified with reference to standard laboratory manuals; guidance from the supervising parasitologist; and their key morphological features, including shape, size, external structures, and coloration [18, 22–24]. Identification was limited to genus‐level classification based on morphology, as molecular techniques were not employed in this study.

### 2.5. Data Analysis

The data were summarized using means, standard deviations, frequencies, and percentages. The characteristics and prevalence of GIP across rearing systems and buffalo groups were examined using information obtained from farm owners, alongside socioeconomic, demographic, farm management, and hygiene‐related variables. Group differences were evaluated using chi‐square tests or Fisher’s exact tests for categorical variables and independent *t* tests for continuous variables. Associations between potential risk factors, including management practices, demographic attributes, hygiene behaviors, feeding behaviors, and contextual conditions, and GIP, were assessed through bivariate and multivariate logistic regression analyses. Variables showing significant associations in the bivariate model (*p* < 0.05) were forcibly entered into the multivariate model for mutual adjustment. This approach was adopted due to the limited sample size and number of outcome events, to reduce the risk of overfitting. Prior to multivariate modeling, correlations among candidate variables were assessed to minimize multicollinearity. Odds ratios (ORs) and adjusted odds ratios (aORs) with corresponding 95% confidence intervals (CIs) are reported, and statistical significance was defined as *p* < 0.05. All analyses were performed using SPSS statistical software.

## 3. Results

Table 2 shows that commercial farms keep mostly older buffaloes than subsistence farms do. The Murrah breed of buffalo, mostly from traders, is common in commercial farms, whereas crossbred and self‐raised buffaloes are common in subsistence farms. Concrete floors and tin roofs with nonedible bedding are common on commercial farms, whereas edible beddings are common on subsistence farms. However, the status of buffaloes (i.e., milking vs pregnant) or grazing or wallowing and the mode of feeding (cut and carry vs graze) practices were not significantly different between commercial farms and subsistence farms. Subsistence farms only indicated tap water as a source of water, but commercial farms indicated other sources as sources of water. More subsistence farms reported free‐range practices than commercial farms did.

**TABLE 2:** Health management features of female buffaloes on commercial and subsistence farms (*n* = 224).

Practices/Features	Commercial (*n* = 112) *n* (%)	Subsistence (*n* = 112) *n* (%)	*p* value	Total *n* (%)
*Health checkup frequency*				
Regularly	14 (12.5)	7 (6.3)	> 0.05^∗^	21 (9.4)
When needed	98 (87.5)	105 (93.75)		202 (90.6)

*Feed anthelmintic within 6 months*				
Yes	10 (8.9)	16 (14.3)	> 0.05^∗^	26 (11.6)
No	102 (91.1)	96 (85.7)		198 (88.4)

*Other free-range animals*				
Yes	56 (50)	68 (60.7)	> 0.05^∗^	124 (55.4)
No	56 (50)	44 (39.3)		100 (44.6)

*Aware of GIP infestation*				
Yes	112 (100)	100 (89.3)	< 0.001^#^	212 (94.6)
No	0 (0)	12 (10.7)		12 (5.4)

*Deworming frequency*				
Every 6 months	21 (20)	4 (3.6)	< 0.001^#^	25 (11.5)
When needed	84 (80)	108 (96.4)		192 (88.5)

*Use of local treatment*				
Yes	2 (1.8)	29 (25.9)	< 0.001^#^	31 (13.8)
No	110 (98.2)	83 (74.1)		193 (86.2)

*Response to sick animals*				
Immediate	112 (100)	49 (43.8)	< 0.001^#^	161 (71.9)
Wait for a few days	0 (0)	63 (56.3)		63 (28.1)

*Treatment procedure*				
Local pharmacist	28 (25)	73 (65.2)	< 0.001^#^	101 (45.1)
Veterinarian doctor	84 (75)	39 (34.8)		123 (54.9)

^∗^Chi‐square test.

^#^Fisher’s exact test when any cell has a count less than 5.

Table 2 shows the farm and health management features of female buffaloes at commercial and subsistence farms. There was no significant difference between commercial farms and subsistence farms in terms of health checkup frequency, anthelmintic administration practices, or the presence of other free‐range animals. However, most of the owners of commercial farms reported knowledge about helminths and the immediate response to buffalo sickness. However, commercial farms mostly depend on local pharmacists for treatment, whereas subsistence farms mostly consult with veterinarian doctors or local methods for the treatment of buffaloes.

**FIGURE 2:**
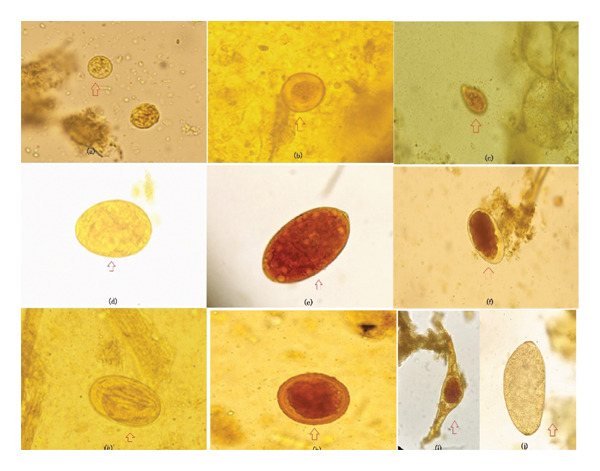
(a) *Entamoeba* spp. (25 μm). (b) Oocyst of coccidian (18 × 12 μm). (c) Trophozoite of *Balantidium* spp. (35 × 65 μm). (d) Egg of *Fasciola* spp. (65 × 48 μm). (e) Egg of *Fasciola* spp. with operculum (65 × 48 μm). (f) Egg of *Haemonchus* spp. (65 × 48 μm). (g) Egg of *Strongyloides* spp. (65 × 30 μm). (h) Egg of *Toxocara* spp. (80 × 65 μm). (i) Egg of *Schistosoma* spp. (185‒45 μm). (j) Egg of *Paramphistomum* spp. (185‒45 μm), up (↑) arrow showing location.

Table 3 shows the prevalence of GIP in female buffaloes at commercial and subsistence farms. A total of 224 fecal samples were evaluated under a microscope via different methods, as described in the methods section. More than half of the samples (i.e., 132 (59%) samples) reported shedding one or more species of GIP (Table 3). Among the 9 species of parasites identified, 3 (i.e., *Entamoeba* spp.*, Balantidium* spp., *and* coccidian) belong to protozoa, 3 to trematodes (i.e., *Fasciola* spp.*, Schistosoma* spp.*, Paramphistomum* spp.), and 3 to nematodes (i.e.*, Strongyloides* spp.*, Toxocara* spp.*, Haemonchus* spp.) (Figure 2). Overall, *Fasciola* spp. had the highest prevalence (30.8%), followed by *Entamoeba* spp. (26.3%) and *Paramphistomum* spp. (12.1%). Compared with those on subsistence farms, the prevalence of nematode parasites on commercial farms was significantly greater (Fisher’s exact test *p* = 0.029) (Table 3). More than one‐third of the samples (38.8%) presented trematode parasites, whereas 29.9% of the samples presented protozoan parasites. Four buffaloes (1.8%) presented with multiple infections, whereas one‐sixth of the samples (i.e., 17%) presented with two infections. Except for overall nematode infections, none of the parasites or categories were significantly different between the farm types. Overall, the subsistence farms presented more protozoan parasites and triple infections, whereas the commercial farms presented more helminth parasites and double infections. Yet, none of these differences were statistically significant. Identification of protozoa and helminths was based solely on morphology; thus, findings—particularly for *Entamoeba* spp., coccidian parasites, and *Toxocara* spp.—should be interpreted with caution and do not imply species‐level confirmation or active infection.

**TABLE 3:** Prevalence of GIP in female buffalo at commercial and subsistence farms (*n* = 224).

Parasite species	Commercial (*n* = 112)	Subsistence (*n* = 112)	Chi‐square *p* value	Total *n* (%)
*Protozoan parasites*				
*Entamoeba* spp.	25 (22.3)	34 (30.4)	> 0.05^∗^	59 (26.3)
*Balantidium* spp.	2 (1.8)	4 (3.6)	> 0.05^#^	6 (2.7)
*Coccidian*	2 (1.8)	1 (0.9)	> 0.05^#^	3 (1.3)
Any protozoan	29 (25.9)	38 (33.9)	> 0.05^∗^	67 (29.9)

*Helminth parasites*				
*Nematodes*				
*Strongyloides* spp.	7 (6.3)	2 (1.8)	> 0.05^#^	9 (4.0)
*Toxocara* spp.	3 (2.7)	1 (0.9)	> 0.05^#^	4 (1.8)
*Haemonchus* spp.	2 (1.8)	0 (0)	> 0.05^#^	2 (0.9)
Any nematodes	12 (10.7)	3 (2.7)	< 0.05^#^	15 (6.7)

*Trematodes*				
*Fasciola* spp.	38 (33.9)	31 (27.7)	> 0.05^∗^	69 (30.8)
*Schistosoma* spp.	0 (0)	1 (0.9)	> 0.05^#^	1 (0.4)
*Paramphistomum* spp.	16 (14.3)	11 (9.8)	> 0.05^∗^	27 (12.1)
Any trematodes	48 (42.9)	39 (34.8)	> 0.05^∗^	87 (38.8)
Any infection	70 (62.5)	62 (55.4)	> 0.05^∗^	132 (58.9)
Single infection	46 (41.1)	46 (41.1)	> 0.05^#^	92 (41.1)
Double infection	23 (20.5)	15 (13.4)	> 0.05^#^	38 (17)
Triple infection	1 (0.9)	3 (2.7)	> 0.05^#^	4 (1.8)

^$^Chi‐square test.

^#^Fisher’s exact test when any cell has a count less than 5.

Table 4 shows the associations between parasitic infections and associated factors. The presence of any GIP was greater among the free‐range female adult buffaloes with reported wallowing, if the owner had other free‐range animals and the owner did not report regular health checks of buffaloes compared with their counterparts (*p* < 0.001) in the univariate model. When all the associated factors in the univariate model were forcibly entered into the multivariate model for mutual adjustment, only free‐range rearing style (aOR = 3.11, 95% CI: 1.15–8.43) and lack of regular checkup practices (aOR = 2.92, 95% CI: 1.05–8.12) remained significant contributing factors for any GIP.

**TABLE 4:** Prevalence and odds ratios of GIP associated with farm practices and buffalo characteristics via logistic regression analysis (*n* = 224).

	%	Univariate OR (95%CI)	Multivariate^∗^ aOR (95%CI)
*Socioeconomic (SES) characteristics*			
*Farm type*			
Commercial	62.5	ref	
Subsistence	55.4	0.74 (0.43–1.27)	

*Breed of buffalo*			
Murrah	68.8	ref	
Crossbred	58.2	0.63 (0.21–1.89)	

*Source of livestock*			
Self‐raised	59.8	ref	
Bought from traders	58.5	0.95 (0.55–1.65)	

*Type of shed*			
Stall barn	58.5	ref	
Loose housing	71.4	1.77 (0.34–9.34)	

*Flooring and roofing of shed*			
Concrete floor and tin roof	59.0	ref	
Earth floor and tin roof	42.9	0.52 (0.11–2.38)	

*Use of bedding*			
Nonedible bedding	60.5	ref	
Edible bedding	55.8	0.82 (0.47–1.44)	

*Present condition of the buffalo*			
Pregnant	57.5	ref	
Milking	60.6	1.14 (0.67–1.94)	

*Free range*			
No	50.6	ref	ref
Yes	77.1	3.29 (1.73 to 6.24)	3.11 (1.15 to 8.43)

*Wallowing*			
No	54.1	ref	ref
Yes	75.0	2.55 (1.27 to 5.11)	0.87 (0.29–2.59)

*Source of water*			
Tap water	59.4	ref	
Other sources	42.9	0.51 (0.11–2.34)	

*Health checkup frequency*			
Regularly	28.6	ref	ref
When needed	62.1	4.09 (1.52 to 10.99)	2.92 (1.05 to 8.12)
*Feed anthelmintic within 6 months*			
Yes	50.0	ref	
No	60.1	1.51 (0.66–3.42)	

*Other free-range animals*			
Yes	51.0	ref	ref
No	65.3	1.81 (1.06 to 3.10)	1.54 (0.87–2.71)

*Aware of GIP infestation*			
Yes	60.4	ref	
No	33.3	0.33 (0.10–1.12)	

*Deworming frequency*			
Every 6 months	52.0	ref	
When needed	59.4	1.35 (0.59–3.11)	

*Use of local treatment*			
Yes	64.5	ref	
No	58.0	0.76 (0.35–1.68)	

*Response to sick animals*			
Immediate	60.9	ref	
Wait for few days	54.0	0.75 (0.42–1.36)	

*Treatment procedure*			
Local pharmacist		ref	
Veterinarian doctor		0.89 (0.52–1.53)	

*Note:* 95% CI: 95% confidence interval, %: prevalence percentage, ref: reference.

Abbreviations: aOR = adjusted odds ratio, OR = odds ratio.

^∗^Model adjusted for all variables, model fit *p* < 0.05, significant, Nagelkerke *R* square: 0.11.

## 4. Discussion

**FIGURE 3:**
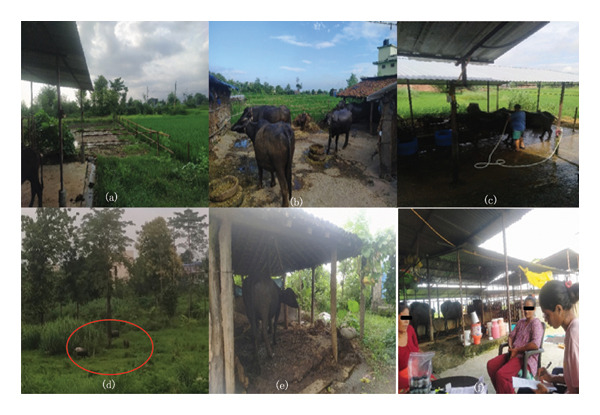
(a) Wallowing area on a commercial farm (Ward 17); (b) loose‐housing method on a commercial farm (Ward 15); (c) farm owner bathing buffaloes on a commercial farm (Ward 13); (d) free‐ranging buffaloes from subsistence farms (Ward 16); (e) subsistence farm with edible bedding (Ward 11); and (f) questionnaire survey with a farm owner (Ward 19).

This study investigated the prevalence of GIPs in adult female buffaloes raised under subsistence and commercial farming systems in Butwal submetropolitan city, Nepal. Overall, 58.9% of buffaloes were infected, a prevalence (i.e., 58.82%) similar to findings from Mahottari and Dhanusha, Nepal [25], and 58.59% prevalence in cows and buffaloes in Pakistan [26]. However, the findings were lower than the 90% prevalence reported from Chitwan, Nepal [27], and 100% in Bangladesh [16]. Conversely, the prevalence was higher than the 39.9% reported in Eastern Nepal [28], and 48.31% reported in Bangladesh [29]. This discrepancy in the prevalence of GIP might be due to differences in climatic conditions, breeds [30], rearing conditions, and levels of awareness [19]. The high prevalence of GIP in our study may be partly attributed to free‐range rearing practices and frequent cross‐contamination during group wallowing, where buffaloes often drink or immerse themselves in muddy, contaminated water. Poorly maintained sheds, characterized by delayed or infrequent dung removal and contamination of feed with fecal material, likely further contributed to parasite transmission (PI AN, personal observation, Figures 3(b), 3(e)). The pattern and severity of parasitic infection can be attributed to the local and microenvironment and conditions in which the animals are kept [31] and poor routine treatment [32]. Furthermore, this relatively high prevalence might be due to poor farmer awareness and a lack of anthelminthic use [26], as well as differences in climate, grazing practices, water sources, hygiene measures, and management techniques [3]. Seasonal fluctuations play a significant role, as monsoon periods create ideal conditions for parasite development and transmission [33]. Grazing practices (i.e., ∼one‐third free range in this study, Figure 3(d)) at the banks of streams and rivers may also be a reason for the relatively higher prevalence in this study, as they provide easy access to intermediate hosts (snails), as trematodes were found to be more prevalent in the study area [3]. Farm management practices such as the use of edible bedding (Figure 3(e)) could also be a contributing factor [29, 34]. Given the purposive nature of farm selection and geographic focus, findings should be interpreted with caution and may not be generalizable to all buffalo‐rearing regions of Nepal. Nevertheless, the study provides valuable preliminary insights into system‐specific risk factors for gastrointestinal parasitism that can inform future, larger‐scale investigations.

The prevalence of GIP among adult female buffaloes in commercial farms (62.5%) was lower than that reported in Pakistan (68.13%) and India (70.45%) [26, 35] but higher than that reported in Italy (33.1%) and Pakistan (29.04%) [9, 36]. Similarly, the prevalence in subsistence farms (55.4%) was higher than that in Poland (44%) and Mexico (42%) [34, 37] but lower than that in Nepal (86%) and Greece (92.73%) [25, 38]. *Fasciola* spp. (30.8%) and *Entamoeba* spp. (26.3%), followed by *Paramphistomum* spp. (12.1%), were the most prevalent parasites on both commercial and subsistence farms. These findings align with previous studies that reported high prevalence rates of *Fasciola* spp. in buffaloes across different regions, including Nepal [39], China [40], and Bangladesh [41]. Similarly, high rates of *Entamoeba* spp. and *Balantidium* spp. infections in buffaloes have been reported in Chitwan, Nepal [27], Mexico [37], and Greece [38]. As shown in Table 4, buffaloes that were more exposed to the free range were more likely to become infected with GIPs. Interestingly, commercial farms had more *Fasciola* spp. infections (33.9%) than did subsistence farms (27.7%). This difference contrasts with similar studies reported from Chitwan, Nepal [27], and southern Nepal [42], where captive farms and commercial farms presented relatively high *Fasciola* spp. infections, which could be because almost half of the buffaloes in this study from commercial farms were free‐ranging doing riverbank grazing, doing wallowing, and were fed with grass from riverbanks. As seen during the study, some commercial farm holders prepare small ponds around the farms so that the buffaloes can wallow during hot weather (Figure 3(a)). Additionally, the high prevalence in some areas may be linked to a lack of deworming programs and poor farm sanitation, as evidenced in studies from Bangladesh [29] and Sri Lanka [43]. A key objective of this study was to compare the GIP burden between commercial and subsistence farms. While there was no statistically significant difference in overall prevalence, commercial farms presented a significantly greater prevalence of nematodes (*p* = 0.029), whereas subsistence farms presented a slightly greater prevalence of protozoan infections. The higher nematode burden on commercial farms may be attributed to the close confinement of a large number of buffaloes, facilitating parasite cross‐transmission [43]. Conversely, higher rates of protozoan infections on subsistence farms could be linked to water contamination and grazing on communal land [44]. These patterns suggest possible system‐specific tendencies, but given the shared use of riverbank grazing and water sources by both commercial and subsistence buffaloes, environmental exposures may have been similar. Additionally, many commercial farms practiced partial free‐ranging practices, which may have further blurred differences in exposure risks. Therefore, any conclusions about the influence of farming systems on infection burden should be interpreted with caution.

This study also revealed significantly higher odds of GIP among free‐ranging adult female buffaloes (aOR = 3.11, 95% CI: 1.15–8.43), regardless of whether the animals grazed in open fields and riverbanks or were allowed to roam within semiconfined areas alongside other domestic livestock. These findings align with results from southern Nepal [42], where free‐range management was similarly associated with elevated parasitic infections. Evidence from Uttar Pradesh, India, also supports this pattern, reporting higher *Fasciola* spp. prevalence among free‐grazing buffaloes exposed to *Lymnaea* snails along riverbanks [45]. Although buffaloes on commercial farms were more confined, many were fed cut‐and‐carry grasses harvested from the same riverbank and field areas where subsistence buffaloes grazed, likely contributing to the high overall GIP burden, particularly via elevated trematode infections. Grazing near water bodies increases the risk of ingesting infective stages of parasites such as *Fasciola* spp., given the abundance of infected *Lymnaea* snails. Additionally, buffaloes that defecate along riverbanks during grazing may further sustain the transmission cycle by depositing parasite eggs into snail‐inhabiting environments. Yet, shared practices and ecological overlaps may explain why the overall infection prevalence did not differ significantly between farming systems, despite some variation in specific parasite groups. Taken together, these findings highlight how local ecological conditions—particularly riverbank grazing, wallowing behavior, and exposure to snail‐rich aquatic environments—interact with management practices to favor trematode transmission. This ecological backdrop helps explain the dominance of *Fasciola* spp. observed in the study area, beyond simple comparisons of prevalence between farming systems.

Multivariate logistic regression analysis also identified the frequency of buffalo health checkups and treatments as a significant risk factor for GIP infections. Irregular or infrequent health monitoring substantially increases parasite burden, as untreated or poorly managed animals are consistently shown to have higher infection rates than those receiving routine veterinary care [26]. A lack of scheduled health assessments is often linked to farmers’ limited awareness of recommended parasite control practices, contributing to heavy parasite loads and clinical issues such as diarrhea, reduced growth, and poor overall conditions [46]. Regular health checks facilitate early diagnosis and timely intervention, enabling strategic deworming, improved pasture management, and nutritional support that collectively reduce both the risk and severity of GIP infections [6]. These findings underscore the need to strengthen farm management practices and promote structured deworming programs to control parasitic infections effectively [27]. Consistent with studies from Malaysia [3] and India [33], limiting unrestricted free‐ranging and ensuring clean water sources may further reduce parasite transmission. These associations should be interpreted as indicative of relevant management‐level risk factors rather than as comprehensive or causal explanations of GIP transmission across farming systems.

The cross‐sectional design restricts the generalizability of our findings. Nevertheless, this study detailed the parasite profile of chosen buffalo farms and examined factors related to GIP in various rearing systems and farm management factors. As patent *Toxocara vitulorum* infections rarely occur in adults, this finding may reflect contamination or passive passage rather than true infection. Selecting subsistence farms in proximity to commercial farms may have reduced environmental heterogeneity and potentially minimized detectable differences attributable to production practices. Although animals were randomly selected within farms, clustering at the farm level was not accounted for in the analysis. This may have resulted in an underestimation of variance and reduced precision of some effect estimates. The lack of a formal sample size calculation is an additional limitation that may affect the generalizability and precision of the findings. The multivariate model demonstrated limited explanatory power, as reflected by a low Nagelkerke *R* ^2^ value. Additionally, variables were selected for multivariate analysis based on bivariate significance, which may have resulted in the omission of relevant confounders not detected in univariate analyses. Although this approach was used to avoid overadjustment, given the small sample size and limited outcome events, residual confounding and reduced model fit cannot be ruled out and should be considered when interpreting the findings. The study was limited to adult female buffaloes (≥ 3 years), reflecting prevailing production practices in the study area, where adult males are rarely maintained due to reliance on artificial insemination and early sale of male calves for meat. While this restriction improved comparability between farming systems, it limits generalizability to male and younger animals. Exclusion of younger buffaloes may have underestimated nematode prevalence, as younger age groups are often more susceptible to GIP infections. Owing to the study’s scope, parasitological and molecular‐level analyses to confirm the zoonotic potential of the identified parasites were not conducted, representing an area for future research to further clarify these aspects.

## 5. Conclusion

This study revealed a high burden of GIP infections in adult female buffaloes across both commercial and subsistence farms in Butwal, Nepal, with an overall prevalence of 58.9%. Trematodes, particularly *Fasciola* spp., were the most dominant parasites, followed by protozoans such as *Entamoeba* spp. Commercial farms presented a higher prevalence of nematodes, which may be influenced by management‐related factors such as periods of confinement and shared feeding spaces, although these mechanisms were not directly assessed in this study. In contrast, subsistence farms showed relatively greater protozoan infections, which may reflect differences in hygiene practices, water sources, and feeding materials rather than confirmed environmental contamination.

Free‐ranging practices and irregular veterinary checkups were identified as significant risk factors, indicating important associations with increased GIP prevalence rather than definitive causal pathways. Although subsistence farms tended to experience higher infection levels potentially related to hygiene limitations, overlapping management practices—including partial free‐ranging and shared exposure environments—may also contribute to transmission dynamics in commercial farms. These findings highlight a complex interplay of management and environmental factors rather than clear system‐level causal differences.

These findings underscore the need for improved farm management practices tailored to local conditions, including clean and nonedible bedding, improved flooring, routine deworming, and strengthened veterinary oversight. Given the economic importance of buffalo farming in Nepal for milk and meat production, implementing region‐specific parasite control strategies remains essential. Collaborative efforts among veterinarians, farmers, and policymakers are therefore critical to reduce the burden of GIP sustainably and to support improvements in buffalo health, productivity, and farm profitability.

## Author Contributions

Arti Neupane contributed to conceptualization and methodology, conducted fieldwork, collected and curated samples and data, performed laboratory investigations, and prepared the original draft. Shristi Bhandari assisted with methodology, parasite identification, data curation, and manuscript review and editing. Tikaram Khanal supported the methodology and investigation, contributed to laboratory analyses and data curation, and assisted with manuscript editing. Kishor Pandey contributed to conceptualization, methodology, investigation, supervision, validation, and resource provision, and participated in manuscript review and revision. Rajendra Prasad Parajuli led conceptualization, methodology, formal analysis, visualization, project administration, supervision, data curation, validation, and resource provision and contributed to manuscript review, editing, and final approval.

## Funding

This study did not receive any financial support from institutions or individuals. The work was conducted as part of the M.Sc. thesis of Mrs. Arti Neupane.

## Ethics Statement

The authorization to collect fecal samples was granted by the Nepal Veterinary Council Ethics Committee (Approval No: 82/2080/81). Informed consent was obtained from all farm owners before sample collection, with assurances of confidentiality, anonymity, and the right to withdraw at any time without consequences. Prior to sampling, the cattle owners were verbally informed about the study’s purpose and reassured that the procedures would not harm their animals. Fecal samples were collected under strict hygienic conditions, and all specimen handling and collection procedures followed relevant institutional and national guidelines. No animals were harmed during the sampling process.

## Conflicts of Interest

The authors declare no conflicts of interest.

## Data Availability

The datasets used during the current study are available from the corresponding author upon reasonable request.

## References

[1] Ministry of Agriculture and Livestock Development , Statistical Information on Nepalese Agriculture (2077/78), Government of Nepal. (2021) 73, Kathmandu, Nepal, 1–26.

[2] National Agriculture and Food Security (NAFS) , Nepal Agriculture and Food Security Country Investment Plan, 2010, Kathmandu: Government of Nepal, https://faolex.fao.org/docs/pdf/nep148989.pdf.

[3] Harizt A. M. , Malahubban M. , Syed-Hussain S. S. et al., Gastrointestinal Parasitic Infections of Buffaloes (Bubalus Bubalis) in Sarawak Borneo: Prevalence, Risk Factors, and Farming Practices, Tropical Biomedicine. (2021) 38, no. 3, 318–326, 10.47665/TB.38.3.072. 34508339

[4] Singh N. K. , Juyal P. D. , Haque M. , and Rath S. S. , Epidemiology of Gastrointestinal Parasites in Buffalo Calves of Punjab State, Journal of Veterinary Parasitology. (2012) 26, no. 1, 19–22.

[5] Rast L. , Lee S. , Nampanya S. , Toribio J. A. L. M. L. , Khounsy S. , and Windsor P. A. , Prevalence and Clinical Impact of Toxocara vitulorum in Cattle and Buffalo Calves in Northern Lao PDR, Tropical Animal Health and Production. (2012) 45, no. 1, 539–546, 10.1007/s11250-012-0256-4. 22945429

[6] Akande F. and Alohutade M. , Diagnosis of Bovine Gastrointestinal Parasites: Comparison of Different Techniques and Different Solutions, Annals of Parasitology. (2021) 67, no. 3, 407–416, 10.17420/ap6703.354. 34953116

[7] Raza M. A. , Prevalence of Intestinal Parasites in Small Ruminants and Their Senstivity to Treatments with Ethnobotanical Remedies in Cholistan, 2013, Pakistan.

[8] Liu Y. , Li F. , Liu W. et al., Prevalence of Helminths in Water Buffaloes in Hunan Province, China, Tropical Animal Health and Production. (2009) 41, no. 4, 543–546, 10.1007/s11250-008-9219-1. 18704740

[9] Khan M. N. , Sajid M. S. , Khan M. K. , Iqbal Z. , and Hussain A. , Gastrointestinal Helminthiasis: Prevalence and Associated Determinants in Domestic Ruminants of District Toba Tek Singh, Punjab, Pakistan, Parasitology Research. (2010) 107, no. 4, 787–794, 10.1007/s00436-010-1931-x. 20532913

[10] Elelu N. and Eisler M. C. , A Review of Bovine Fasciolosis and Other Trematode Infections in Nigeria, Journal of Helminthology. (2018) 92, no. 2, 128–141, 10.1017/S0022149X17000402. 28528590

[11] Raza M. A. , Iqbal Z. , Jabbar A. , and Yaseen M. , Point Prevalence of Gastrointestinal Helminthiasis in Ruminants in Southern Punjab, Pakistan, Journal of Helminthology. (2007) 81, no. 3, 323–328, 10.1017/s0022149x07818554. 17711599

[12] Roberts A. A. and Fernando J. A. , The Significance of the Gastrointestinal Parasites of Asian Buffalo in Sri Lanka, Veterinary Research Communications. (1990) 14, no. 6, 481–488, 10.1007/bf00367060. 2284708

[13] Khan T. , Nasreen N. , Shater A. F. et al., Risk Factor Analysis for the Prevalence of Gastrointestinal Parasites Found in Large Ruminants in Lower Dir Khyber Pakhtunkhwa Pakistan, Saudi Journal of Biological Sciences. (2021) 28, no. 12, 7022–7026, 10.1016/j.sjbs.2021.07.078. 34867003 PMC8626251

[14] Alam M. R. , Erfan R. , Sen A. B. , Das S. , Rahman M. , and Nath S. K. , Prevalence of Gastrointestinal Helminthiasis in Naturally Infested Buffalo in Sylhet District, International Journal of Advanced Multidisciplinary Research. (2016) 3, no. 8, 52–58.

[15] Shaw D. J. , Vercruysse J. , Claerebout E. , Agneessens J. , and Dorny P. , Gastrointestinal Nematode Infections of first-season Grazing Calves in Belgium: General Patterns and the Effect of Chemoprophylaxis, Veterinary Parasitology ELSEVIER Veterinary Parasitology. (1997) 69, no. 2, 103–116, 10.1016/s0304-4017(96)01105-3. 9187035

[16] Roy P. P. , Begum N. , Dey A. R. , Sarker S. , Biswas H. , and Farjana1 T. , Prevalence of Gastrointestinal Parasites of Buffalo at Mongla, Bagerhat, International Journal of Natural and Social Sciences. (2016) 3, no. 1, 59–66.

[17] Central bureau of statistics (CBS) , National Population and Housing Census 2021, 2021, Citeseer.

[18] Soulsby E. J. L. , Helminths, Arthropods and Protozoa of Domesticated Animals, 1982.

[19] Adhikari R. B. and Ghimire T. R. , A Case Study of Multiple Parasitisms in a Calf Buffalo (Bubalus Bubalis), Agricultural Science Digest-A Research Journal. (2021) 41, no. spl, 237–241, 10.18805/ag.d-5172.

[20] Beaver P. C. , Helminths, Arthropods and Protozoa of Domesticated Animals, The American Journal of Tropical Medicine and Hygiene. (2017) 32, no. 4, 10.4269/ajtmh.1983.32.906.

[21] Dhakal P. , Sharma H. P. , Shah R. , Thapa P. J. , and Pokheral C. P. , Copromicroscopic Study of Gastrointestinal Parasites in Captive Mammals at Central Zoo, Lalitpur, Nepal, Veterinary Medicine and Science. (2023) 9, no. 1, 457–464, 10.1002/vms3.1039. 36495198 PMC9857001

[22] Foreyt W. J. , Veterinary Parasitology Reference Manual, 2013, John Wiley & Sons.

[23] Parija S. C. and Chaudhury A. , Textbook of Parasitic Zoonoses, 2022, Springer.

[24] Zajac A. M. , Conboy G. A. , Little S. E. , and Reichard M. V. , Veterinary Clinical Parasitology, 2021, John Wiley & Sons.

[25] Yadav S. K. , Epidemiological Survey of Fascioliasis in Cattle, Buffalo and Goat in Mahottari and Dhanusha, Nepal, The Journal of Advances in Parasitology. (2015) 2, no. 3, 52–56, 10.14737/journal.jap/2015/2.3.52.56.

[26] Khan T. , Khan W. , Iqbal R. , Maqbool A. , Fadladdin Y. A. J. , and Sabtain T. , Prevalence of Gastrointestinal Parasitic Infection in Cows and Buffaloes in Lower Dir, Khyber Pakhtunkhwa, Pakistan, Brazilian Journal of Biology. (2023) 83, 1–6, 10.1590/1519-6984.242677. 35137844

[27] Adhikari R. B. , Adhikari Dhakal M. , and Ghimire T. R. , Prevalence and Diversity of Gastrointestinal Parasites in Domestic Buffaloes (Bubalus Bubalis linnaeus, 1758) Reared Under Captive and Semi-captive Conditions in Ratnanagar, Chitwan, Nepal, Annals of Parasitology. (2022) 68, no. 4, 701–713, 10.17420/ap6804.477. 37702207

[28] Sah R. P. , Prasai H. K. , Shrestha J. , Talukder M. H. , Rahman A. A. , and Sah R. B. , Seasonal and Altitudinal Prevalence of Fascioliasis in Buffalo in Eastern Nepal, Journal of Nepal Agricultural Research Council. (2018) 4, 48–53, 10.3126/jnarc.v4i1.19689.

[29] Mamun M. A. A. , Begum N. , and Mondal M. M. H. , A Coprological Survey of gastro-intestinal Parasites of Water Buffaloes (Bubalus Bubalis) in Kurigram District of Bangladesh, Journal of the Bangladesh Agricultural University. (2011) 9, no. 1, 103–109, 10.3329/jbau.v9i1.8752.

[30] Khanal M. and Subedi J. R. , Prevalence of Gastrointestinal Parasites in Pigs (Sus Domesticus Linnaeus, 1758) of Chandragiri Municipality, Kathmandu, Nepal, Journal of Animal Science and Veterinary Medicine. (2020) 5, no. 2, 48–55, 10.31248/jasvm2020.195.

[31] Maganga G. D. , Kombila L. B. , Boundenga L. et al., Diversity and Prevalence of Gastrointestinal Parasites in Farmed Pigs in Southeast Gabon, Central Africa, Veterinary World. (2019) 12, no. 12, 1888–1896, 10.14202/vetworld.2019.1888-1896. 32095037 PMC6989316

[32] Omoruyi Z. and Agbinone I. , Gastrointestinal Parasites Among Swine Bred in Edo State, Nigeria, African Journal of Clinical and Experimental Microbiology. (2020) 21, no. 4, 349–353, 10.4314/ajcem.v21i4.12.

[33] Marskole P. , Verma Y. , Dixit A. K. , and Swamy M. , Prevalence and Burden of Gastrointestinal Parasites in Cattle and Buffaloes in Jabalpur, India, Veterinary World. (2016) 9, no. 11, 1214–1217, 10.14202/vetworld.2016.1214-1217. 27956771 PMC5146300

[34] Kobak P. and Pilarczyk B. , Prevalence of Gastrointestinal Parasites of Water Buffaloes Raised in the Notecka Forest Region (Poland), 2012, 33–36, 10.2478/v10213-012-0006-4.

[35] Renwal K. K. , Gupta A. , Kumar N. , Pilania P. K. , and Manohar G. S. , Prevalence and Risk Assessment of Gastrointestinal Helminthoses in Dairy Animals of Bikaner, Rajasthan, Journal of Parasitic Diseases. (2017) 41, no. 2, 557–561, 10.1007/s12639-016-0850-x. 28615878 PMC5447627

[36] Condoleo R. U. , Veneziano V. , Bruni G. et al., Distribution of Helminths in Buffalo Farms from Central Italy Distribution of Helminths in Buffalo Farms from Central Italy, 2016, 10.4081/ijas.2007.s2.920.

[37] Ojeda-Robertos N. F. , Torres-Chablé O. M. , Peralta-Torres J. A. et al., Study of Gastrointestinal Parasites in Water Buffalo (Bubalus Bubalis) Reared Under Mexican Humid Tropical Conditions, Tropical Animal Health and Production. (2017) 49, no. 3, 613–618, 10.1007/s11250-017-1237-4. 28161847

[38] Founta A. , Papadopoulos E. , Chliounakis S. , Bampidis V. A. , and Papazahariadou M. , Presence of Endoparasites in the Greek Buffalo (Bubalus Bubalis) from Northern Greece, Journal of the Hellenic Veterinary Medical Society. (2018) 69, no. 2, 999–1003, 10.12681/jhvms.18019.

[39] Joship B. R. and Mahato S. N. , Gastrointestinal Parasitic Diseases of Buffaloes and Implications of Climate Change for These Diseases in Nepal, 2013.

[40] Zhang J. L. , Si H. F. , Zhou X. Z. , Shang X. F. , Li B. , and Zhang J. Y. , High Prevalence of Fasciolosis and Evaluation of the Efficacy of Anthelmintics Against Fasciola Hepatica in Buffaloes in Guangxi, China, International Journal of Parasitology: Parasites and Wildlife. (2019) 8, 82–87, 10.1016/j.ijppaw.2018.12.010. PMC6330380 30671343

[41] Saha S. , Bhowmik D. , and Chowdhury M. , Prevalence of Gastrointestinal Helminthes in Buffaloes in Barisal District of Bangladesh, Bangladesh Journal of Veterinary Medicine. (2014) 11, no. 2, 131–135, 10.3329/bjvm.v11i2.19137.

[42] Patel D. K. , Subedi J. R. , Dhakal P. , and Parajuli R. P. , Intestinal Parasitic Infections (IPIs) and Contributing Factors in Bovine Calves in Southern Nepal, Veterinary Medicine and Science. (2025) 11, no. 2, 10.1002/vms3.70254. PMC11843029 39980474

[43] Gunathilaka N. , Niroshana D. , Amarasinghe D. , and Udayanga L. , Prevalence of Gastrointestinal Parasitic Infections and Assessment of Deworming Program Among Cattle and Buffaloes in Gampaha District, Sri Lanka, BioMed Research International. (2018) 2018, 1–10, 10.1155/2018/3048373. PMC6198547 30402469

[44] Mamunur Rashid M. , Zamal Uddin M. , Zakir Hossain M. et al., Agriculture, Livestock and Fisheries Status of Intestinal Schistosomiasis in Buffaloes and Cattle in Rajshahi, Bangladesh Article Info Abstract, Research Article Res. Agric. Livest. Fish. (2022) 9, no. 2.

[45] Gupta S. C. , Ghosh S. , Raina O. K. et al., Status and Prevalence of Fasciolosis in Cattle and Buffaloes in Different agro-climatic Zones of Uttar Pradesh, India, Journal of Veterinary Parasitology. (2008) 22, no. 2, 59–63.

[46] Yousaf A. , Prevalence of Gastrointestinal Parasites in Buffalo and Cow Calves in Rural Areas of Rawalpindi, Pakistan, Biomedical Journal of Scientific & Technical Research. (2021) 40, no. 2, 32159–32165, 10.26717/bjstr.2021.40.006437.

